# Eumycetoma versus actinomycetoma: Diagnosis on cytology

**DOI:** 10.4103/0970-9371.73297

**Published:** 2010-10

**Authors:** Nishat Afroz, Nazoora Khan, Farhan A Siddiqui, Mehar Rizvi

**Affiliations:** Department of Pathology, J N Medical College, AMU Aligarh, India; 1 Department of Microbiology, J N Medical College, AMU Aligarh, India

**Keywords:** Eumycetoma, fine needle aspiration cytology, *Madurella mycetomatis*, actinomycetoma, culture

## Abstract

Eumycetoma is a chronic cutaneous and subcutaneous infection caused by various genera of fungi producing specific colored granules known as grains. A 45-year-old farmer presented clinically with a left foot mass with multiple discharging sinuses existing for last 3 years. Clinical and radiological findings suggested a diagnosis of chronic osteomyelitis with suspicion of tuberculosis. Imprints plus fine needle aspiration cytology (FNAC) smears exhibited distinct brown-black colonies of a fungus having branching and septate hyphae embedded in matrix like material against a mixed inflammatory background. Periodic acid Schiff (PAS) stain gave positive staining and subsequent fungal culture confirmed the cytological diagnosis and aided in species identification as *Madurella mycetomatis*. Thus, eumycetoma can precisely be diagnosed and confidently differentiated from similar conditions such as actinomycetoma by simple and inexpensive cytological techniques such as FNAC and imprint smears, employing routine May-Grünwald-Giemsa, Papanicolaou and simple PAS stains on cytological specimen, thus leading to rapid diagnosis for institution of correct treatment.

## Introduction

Eumycetoma is a chronic cutaneous and subcutaneous infection caused by various genera of fungi, leading to progressive destruction of soft tissue and the nearby anatomical structures. It is mainly a disease of the tropical and subtropical zones, especially between the Tropic of Cancer and the Tropic of Capricorn, that is, between latitudes 15°S and 30°N. Mycetoma was first described in the mid-19^th^ century and initially named “Madura foot”, after Madurai in India, where the disease was first identified.[1,2] It is endemic in India, Pakistan, parts of Africa, Central and South America and Indonesia.[3] This disease is defined by a triad of tumefaction of the affected tissues, formation of multiple draining sinuses and the presence of grains. It is confirmed serologically, histologically or by culture studies. Most of the reports mainly discuss the histopathological characteristics of mycetoma and only a few studies have described their cytological features.[4,5]

## Case Report

A 45-year-old man, farmer by occupation, presented clinically with a slow growing, slightly tender, firm mass on the plantar aspect of left foot for 3 years, measuring 5×4×3 cm. There were multiple discharging sinuses covered with blood mixed purulent exudates. Radiograph showed features of osteomyelitis and soft tissue shadow. A tentative clinical diagnosis of tuberculous osteomyelitis was proposed. Imprint smears were made from the discharge and fine needle aspiration cytology (FNAC) was done using a 23-gauge needle, attached to a 10-ml syringe. Aspirate consisted of sero-sanguinous, pus like material. Alcohol fixed smears were stained with Papanicolaou (Pap stain), while air-dried smears were stained with May-Grünwald-Giemsa (MGG) stain. Simultaneously, Periodic acid Schiff (PAS), Gram’s stain and acid fast bacilli (AFB) stains were performed. Additionally, fungal and bacterial cultures were carried out.

**Figure 1:**
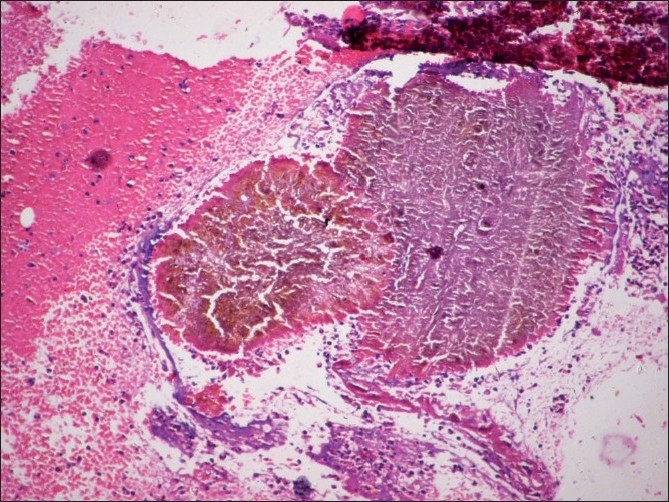
FNAC smear showing brown-black granules amidst inflammatory background (Pap, ×100)

**Figure 2:**
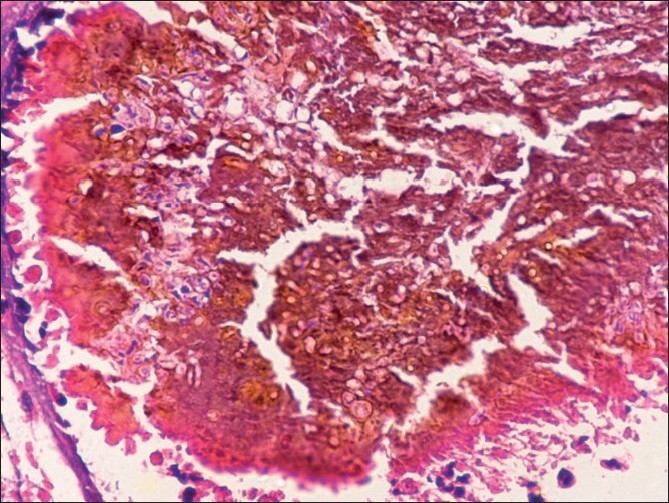
FNAC smear showing branching septate hyphae embedded in a cement-like material (Pap, ×200)

**Figure 3:**
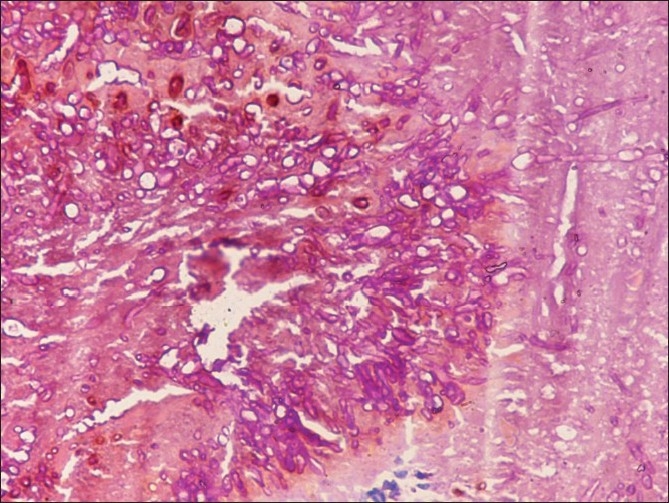
FNAC smear showing PAS positive fungal hyphae (PAS, ×200)

Both FNAC smears and imprint smears comprised plenty of pus cells, some lymphocytes, histiocytes and foreign body giant cells in a necrotic background. Several brown to black colonies were seen. On higher magnification, these consisted of septate, branching fungal hyphae embedded in a cement-like matrix [Figures 1 and 2]. PAS stain highlighted the branching hyphae [Figure 3]. AFB and Gram’s stains were negative. The cytological diagnosis of eumycotic mycetoma was rendered with suggestion of Madura foot. The excised surgical specimen confirmed the diagnosis of eumycetoma. The fungal species was identified as *Madurella mycetomatis* on subsequent fungal culture.

## Discussion

Agents that cause eumycetoma are primarily saprophytic micro-organisms that are found in the soil and on plant matter. Healthy persons become inoculated with these agents as a result of the traumatic implantation of thorns, splinters and other plant matter. Therefore, the incidence of mycetoma is more particularly occupation dependent as people like farmers, herdsmen and field workers are more likely to come in contact with the causative agents.[4] The disease is commonly seen in adult men, with a male to female ratio of 3.5:1.[6] The most common site is foot as 70% of all myetomas affect the foot,[3] hence the name Madura foot. However, extrapedal involvement also occurs and has been detected in hand, leg, head and neck, abdominal wall, buttock and perineum.[6]

Eumycetoma may present as a small localised tumor like mass, with or without sinuses, or can be associated with significant morbidity in terms of gradual enlargement, destruction and deformity of the affected site. The diagnosis of eumycetoma is made tentatively clinically when discharging grains are visible to the naked eye.[6] The grains vary in color, size and consistency depending on the causative agent and can be confirmed by culture method. Granules of eumycetoma are firm 0.2–5 mm aggregates of organised vegetative, septate hyphae, which often are embedded in a matrix cement substance of the eumycetoma, producing black granules. *M. mycetomatis* accounts for most cases worldwide. *Pseudoallescheria boydii* is the common aetiologic agent in the United States, while *Madurella grisea* is a common aetiologic agent in South America.[3] In general, the geographical distribution of the various mycetoma agents is related to the amount of rainfall and other climatic conditions, and thus, each geographical region has a different list of most common agents.

The smears from eumycetoma lesions have a distinct cytological appearance, characterised by (brown to black) colonies of branching, septate (distinct) hyphae embedded in a matrix which stain positively with PAS or Gomori’s methenamine silver stains, both demonstrating large sized hyphae of eumycetoma.[7,8] The differentiation of mycetoma into eumycetoma and actinomycetoma is important as the latter is more amenable to medical treatment than is eumycetoma. The distinction between eumycetoma and actinomycetoma in FNAC is as accurate as histopathology.[6] On hemotoxylin and eosin (H and E) staining, the grains of actinomycetoma appear homogenously eosinophilic, while these appear blue in the centre with pink filaments on the periphery on MGG staining. The grains are also Gram positive.[4,8] Hag *et al*.[4] and Gabhane *et al*.[5] in their case studies have also found similar cytological findings which aided in diagnosis and also in differentiating eumycetoma from actinomycetoma. Thus, to conclude, as observed in our study, a diagnosis of eumycetoma should be suspected in case of discharging sinuses, especially those exhibiting black granules. The cytological diagnosis of eumycetoma can be as accurate as histological diagnosis, and techniques such FNAC as well as imprint smears can definitely be taken into consideration before planning any medical or surgical treatment as these are simple, inexpensive and fairly reliable techniques without any obvious disadvantages. Special fungal stains can also be well applied to cytological specimens for further confirmation, whereas culture studies are helpful in confirmation of diagnosis and species identification.
